# Novel Withanolides from *Tubocapsicum anomalum* Suppress Triple-Negative Breast Cancer by Triggering Apoptosis and p53-ASCT2-SLC7A11-Mediated Ferroptosis

**DOI:** 10.3390/molecules29081838

**Published:** 2024-04-18

**Authors:** Lili Huang, Yingying Wei, Maowei Ni, Hongtao Hu, Luyi Xi, Chen Wang, Zhihui Zhu, Bo Yang, Huajun Zhao

**Affiliations:** 1 School of Pharmaceutical Sciences, Zhejiang Chinese Medical University, Gaoke Rd., Hangzhou 311402, China; 202111014011052@zcmu.edu.cn (L.H.);; 2 The Cancer Hospital of the University of Chinese Academy of Sciences (Zhejiang Cancer Hospital), Institute of Basic Medicine and Cancer (IBMC), Chinese Academy of Sciences, Hangzhou 310022, China; 3 Academy of Chinese Medical Sciences, Zhejiang Chinese Medical University, Binwen Rd., Hangzhou 310053, China

**Keywords:** *Tubocapsicum anomalum*, TNBC, ferroptosis, apoptosis, p53-ASCT2-SLC7A11 axis

## Abstract

Triple-negative breast cancer (TNBC) is a malignant breast cancer. There is an urgent need for effective drugs to be developed for TNBC. *Tubocapsicum anomalum* (*T. anomalum*) has been reported to have an anti-tumor effect, and six novel withanolides were isolated from it and designated as TAMEWs. However, its anti-TNBC effect is still unknown. The results of an MTT assay indicated a higher sensitivity of TNBC cells to TAMEWs compared to other cells. TAMEWs induced apoptosis via mitochondrial dysfunction. They caused increased levels of lipid ROS and Fe^2+^, with downregulation of GSH and cystine uptake, and it has been confirmed that TAMEWs induced ferroptosis. Additionally, the results of Western blotting indicate that TAMEWs significantly decrease the expressions of ferroptosis-related proteins. Through further investigation, it was found that the knockdown of the p53 gene resulted in a significant reversal of ferroptosis and the expressions of its associated proteins SLC7A11, ASCT2, and GPX4. In vivo, TAMEWs suppressed TNBC growth with no obvious damage. The IHC results also showed that TAMEWs induced apoptosis and ferroptosis in vivo. Our findings provide the first evidence that TAMEWs suppress TNBC growth through apoptosis and ferroptosis.

## 1. Introduction

Breast cancer (BC) remains the most widespread malignant tumor among women worldwide. Triple-negative BC (TNBC) is the most aggressive subtype of BC, with negative expression levels of the progesterone receptor, estrogen receptor, and human epidermal growth factor receptor 2. TNBC accounts for approximately 15–20% of all BC cases. There are currently no effective drugs or therapies designed to cure TNBC [1]. Conventional treatments for TNBC, which include chemotherapy, radiotherapy, targeted therapy, endocrine therapy, and surgery, are unable to provide a good prognosis or quality of life for patients [2,3]. Therefore, there is an urgent need to develop effective treatments or drugs for TNBC.

*Tubocapsicum anomalum* (Franch. et Sav.) Makino is a medicinal plant belonging to the Solanaceae family. It is widely distributed in China, including in the regions of Fujian, Guangdong, Guizhou, and Zhejiang. The plant is enriched in withanolides, which are naturally occurring C28 steroids based on an ergostane skeleton and are primarily found in the Solanaceae family [4]. The medicinal use of *T. anomalum* is recorded in ancient Chinese texts; it was described as “heat-clearing and detoxicating” in the *BenCaoShiYi*. The ancient records prompted investigations of TAMEWs in the treatment of TNBC. Traditionally, *T. anomalum* has been used as a folk medicine to treat edema, swelling, and sores. Recent studies have shown that *T. anomalum* exhibits various biological activities, including anti-tumor activity [5,6,7]. Recently, there have been several studies reporting the anti-tumor effects of withanolides, the mechanisms were related to autophagy, ferroptosis, apoptosis, and so on [8,9]. Our group has been dedicated to the precise discovery of natural medicines from herbs. This research has included chemical studies on *T. anomalum*, using N-acetyl cysteine as a pharmacophore probe to search for new compounds that have proven to be highly effective against TNBC and other types of tumors [10]. However, the mechanisms of the effects of *T. anomalum* against TNBC remain unclear.

In this study, a novel fraction of *T. anomalum* comprising six withanolides (*Tubocapsicum anomalum* (Franch. et Sav.) Makino electrophilic withanolides, TAMEWs) was attained. We investigated the anti-tumor activities of TAMEWs and revealed the potential mechanisms of the ferroptosis and apoptosis that are induced by TAMEWs. The findings could be valuable for the future clinical application of TAMEWs in the treatment of TNBC.

Apoptosis is a programmed cell death process. Caspases play a key role in apoptosis by acting as both initiators and executioners of the process. Caspases can be activated through two common pathways, including the intrinsic (or mitochondrial) and extrinsic (or death receptor) pathways [11]. Mitochondria is one of the key organelles of apoptosis. When apoptosis occurs, the mitochondrial membrane potential and permeability will change [12]. During the process of apoptosis, cleaved caspase 3 can cleave various downstream substrates, such as PARP, ultimately leading to significant morphological changes in apoptotic cells [13]. Emerging evidence shows that the combination of ferroptosis and apoptosis enhances the lethality of cancer cells [14]. Ferroptosis is a novel iron-dependent form of cell death driven by polyunsaturated fatty acid peroxidation. Ferroptosis is structurally different from apoptosis, autophagy, and necrosis because it is initiated by the excessive accumulation of lethal lipid reactive oxygen species (ROS) [15,16]. Ferroptosis influences several cancers, such as BC, hepatocellular carcinoma, ovarian cancer, and others [17,18,19]. System Xc^−^ comprises a light chain (solute carrier family 7 member 11, SLC7A11) and a heavy chain (SLC3A2). SLC7A11 (also known as xCT) is overexpressed in multiple cancers, including TNBC [20]. SLC7A11 is a glutamate/cystine antiporter that transports cystine into cells. It has an important role in inhibiting ROS accumulation and hindering the process of ferroptosis [21]. Alanine, serine, and cysteine transporter 2 (ASCT2, also termed SLC1A5) are membrane-localized proteins that act as transporters of glutamine. ASCT2 may have an important role in ferroptosis [22]. Glutamine transferred into cells by ASCT2 can be converted to glutamate when catalyzed by glutaminase 2 (GLS2). Glutamate is then transferred to the extracellular space by SLC7A11 [23]. ASCT2 expression has been related to poor prognosis and overall survival in many cancers, such as hepatocellular carcinoma [24], lung cancer [25], and TNBC [26]. SLC7A11 and ASCT2 both play key roles in amino acid metabolism in ferroptosis.

**Figure 1:**
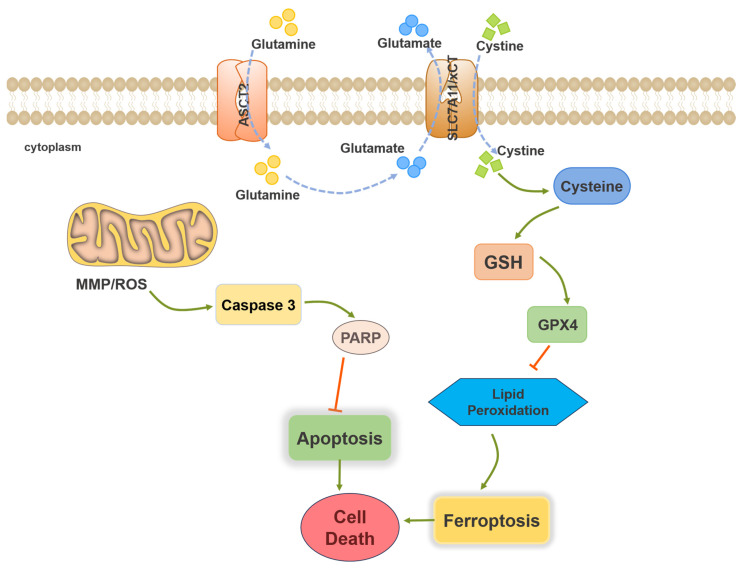
Core molecular mechanisms of ferroptosis and apoptosis.

In general, the crucial function of the glutathione peroxidase 4 (GPX4) is to effectively reduce membrane lipid hydroperoxides (LOOHs) to nontoxic lipid alcohols (LOHs) by utilizing glutathione (GSH) as a co-substrate and reducing agent. This activity protects cells from uncontrolled membrane damage. Inhibiting the activity or decreasing the intracellular levels of GPX4 can induce ferroptosis. GSH participates in ferroptosis by acting as a reducing substrate of GPX4 to mitigate the accumulation of lipid peroxide and protect cells from oxidative damage [27,28]. GPX4 is considered to be the most important protein in ferroptosis. Inhibiting the activity of GPX4 could be an effective method to induce ferroptosis (Figure 1).

## 2. Results and Discussion

### 2.1. TAMEWs Suppresses the Viability of TNBC Cells In Vitro

**Figure 2:**
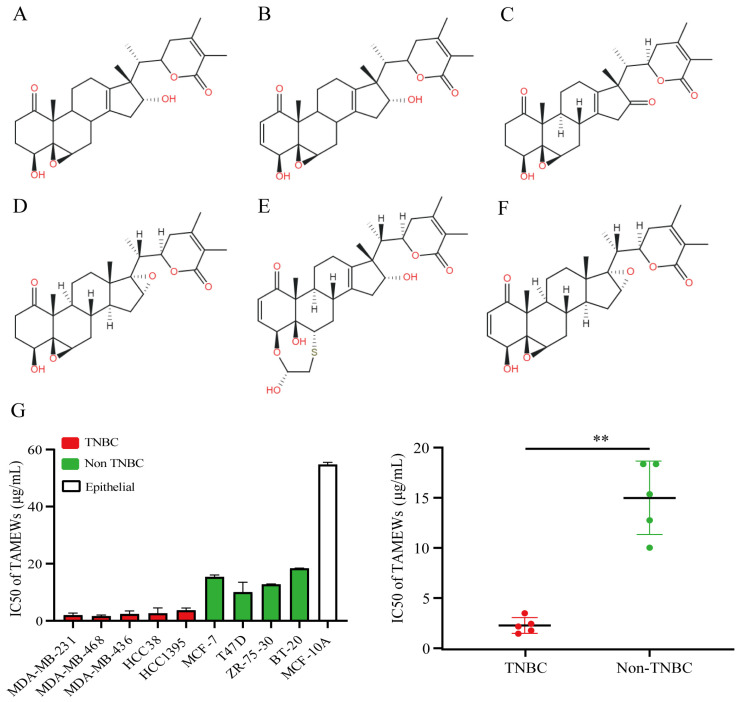
Structures and anti-tumor effects of TAMEWs. (A-F) Structures of the six constituent compounds of TAMEWs. (**A**) Tubocapsenolide B; (**B**) Tubocapsenolide A; (**C**) 16-carbonyltubocapsenolide B; (**D**) Tubocapsenolide H; (**E**) 2,3-Dihydrotubocapsanolide A; (**F**) Tubocapsanolide A. (**G**) The IC_50_ (μg/mL) values of TAMEWs on the viability of TNBC cells, breast cancer cells, and healthy mammary gland epithelial cells were detected by MTT assay. The data are expressed as mean ± SD (*n* = 3). ** *p* < 0.01, compared to the control groups.

Our team has published related research on the chemical composition analysis of *T. anomalum* [10]. A novel fraction consisting of six withanolides was identified from *T. anomalum* and these were designated as TAMEWs (Figure 2A–F). The UPLC-Q-TOF-MS/MS analysis of TAMEWs is shown in Figure S1A,B, and the six components were identified according to the standards of UPLC (Figure S1C). The effects of TAMEWs on the viability of BC cells were determined using the MTT assay. The results found that TAMEWs significantly inhibited the growth of a variety of BC cells after 48 h. The inhibition was more pronounced for TNBC cells than for non-TNBC cells; the inhibition of MCF-10A cells was weak (Figure 2G).

### 2.2. TNBC Cell Inhibition by TAMEWs Might Be via Apoptosis and the Ferroptosis Pathway

**Figure 3:**
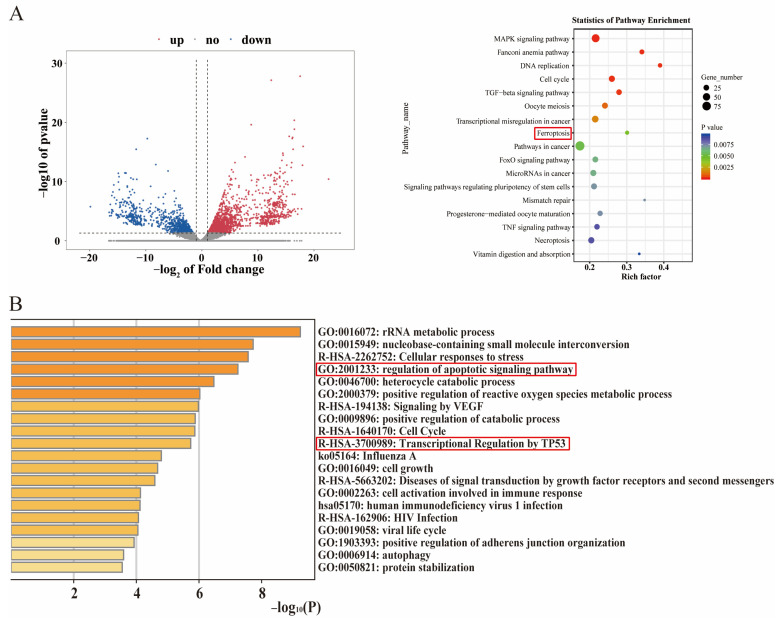
RNA−sequencing (RNA−seq) and proteomic analysis. (**A**) Pathway analysis of the differentially expressed genes using the Metascape web−based platform; volcano plot of down−regulated and upregulated proteins in the TAMEWs−treated group. (**B**) Pathway analysis of the differentially expressed proteins using the Metascape web-based platform. The data are expressed as mean ± SD (*n* = 3).

To clarify the potential molecular mechanisms of TNBC cell death induced by TAMEWs, transcriptomic results showed that the intervention of TAMEWs was associated with ferroptosis (Figure 3A). Label-free proteomic results showed that the anti-tumor effect of TAMEWs was related to the apoptosis signaling pathway and the p53 signaling pathway (Figure 3B). Consequently, the subsequent experiments focused on apoptosis and ferroptosis to clarify the mechanisms by which TAMEWs inhibited TNBC.

### 2.3. TAMEWs Induce Apoptosis via Mitochondrial Dysfunction in TNBC Cells

**Figure 4:**
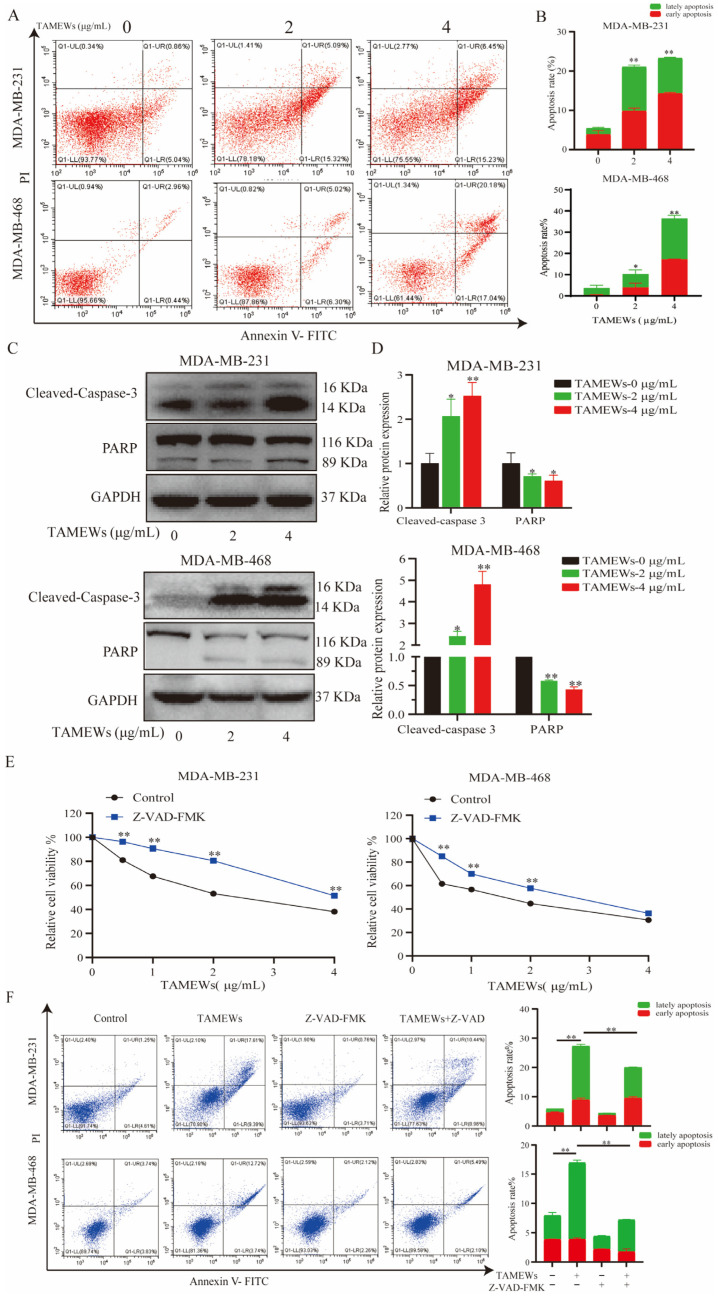
TAMEWs induce apoptosis in TNBC cells. (**A**) TNBC cells were exposed to TAMEWs with indicated concentrations for 24 h, apoptotic cells were stained with Annexin V−FITC/PI and analyzed by flow cytometry. (**B**) Statistical results of the relative apoptosis rates of each group. (**C**) Western blotting assay was performed to detect caspase−related proteins. (**D**) Statistical results of the relative protein expressions of cleaved−caspase 3 and PARP. (**E**) The relative cell viability after the combination of TAMEWs with apoptosis inhibitor Z−VAD−FMK (10 μM) for 48 h. (**F**) Apoptotic cells were stained with annexin V-FITC/PI and analyzed by flow cytometry. The data are expressed as mean ± S.D (*n* = 3). * *p* < 0.05, ** *p* < 0.01 compared to the control groups.

**Figure 5:**
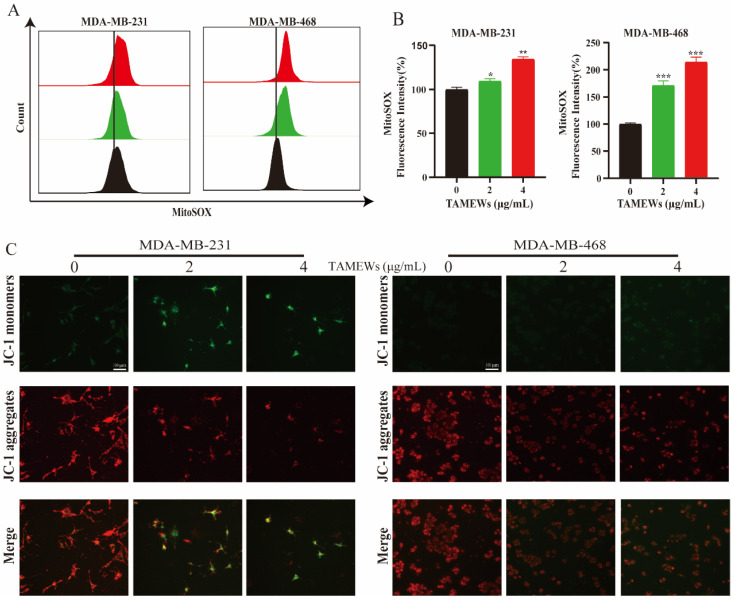
TAMEWs induce apoptosis via mitochondrial dysfunction. (**A**) Mito-ROS was detected by the MitoSOX and analyzed by flow cytometry after treatment with TAMEWs for the indicated concentrations. (**B**) Statistical results of the relative level of mito-ROS in each group. (**C**) The MMP of TNBC cells were stained with JC-1 and analyzed by fluorescence microscope (200× magnification). The data are expressed as mean ± SD (*n* = 3). * *p* < 0.05, ** *p* < 0.01, *** *p* < 0.001 compared to the control groups.

The proteomic analysis implied the apoptosis pathway as the target of the TNBC cell death induced by TAMEWs. A flow cytometry analysis of the apoptotic cells stained with Annexing V-FITC-PI showed that both the MDA-MB-231 and MDA-MB-468 cell lines treated with TAMEWs demonstrated significantly increased apoptosis rates compared to that of control, thus confirming that TAMEWs could induce apoptosis in TNBC cells (Figure 4A,B). Western blotting revealed that caspase 3 and PARP were activated by TAMEW treatment (Figure 4C,D). The apoptosis inhibitor Z-VAD-FMK was used to evaluate the sensitivity of TAMEWs. Z-VAD-FMK partially rescued cell death caused by TAMEWs (Figure 4E). Flow cytometry demonstrated that Z-VAD-FMK could reverse the induction of apoptosis (Figure 4F). Additionally, the flow cytometry results showed that mitochondrial ROS (mito-ROS) was also increased by TAMEW treatment (Figure 5A,B). Immunofluorescence results revealed the loss of the MMP of TNBC cells, evident by the gradual decrease in the level of the JC-1 polymer and the gradual increase in the level of the JC-1 monomer (Figure 5C). The collective findings indicated that apoptosis induced by TAMEWs might result from mitochondrial dysfunction.

### 2.4. TAMEWs Induce Ferroptosis by Regulating Amino Acid Metabolism in TNBC Cells

**Figure 6:**
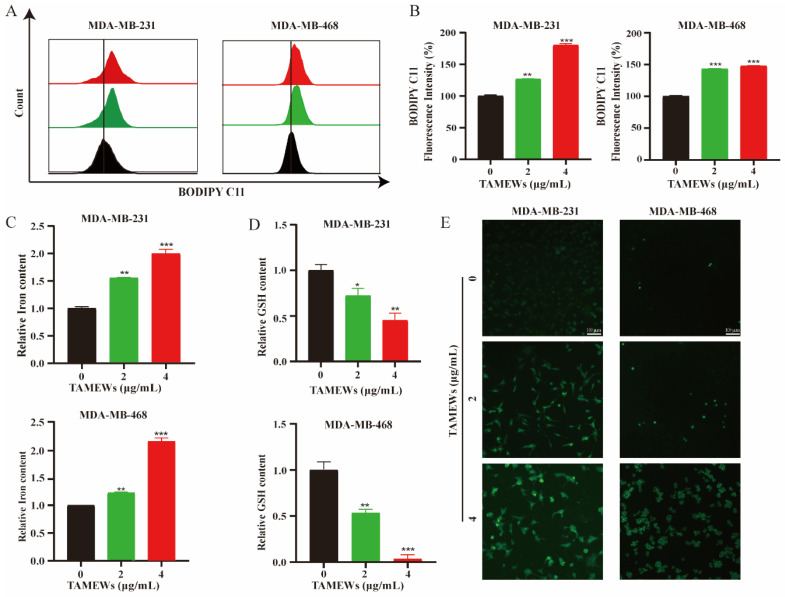
TAMEWs induce ferroptosis in TNBC cells. (**A**) Lipid peroxidation was detected by the BODIPY-C11 (581/591) probe and analyzed by flow cytometry after treatment with TAMEWs at the indicated concentrations. (**B**) Statistical results of the relative level of lipid peroxidation in each group. (**C**) Redox-active iron accumulation was detected by the Iron Assay Kit. (**D**) GSH depletion was detected by the GSH/GSSG Assay Kit. (**E**) Cellular lipid ROS (green) level was observed by fluorescence microscopy (200 × magnification). The data are expressed as mean ± SD (*n* = 3). * *p* < 0.05, ** *p* < 0.01, *** *p* < 0.001 compared to the control groups.

**Figure 7:**
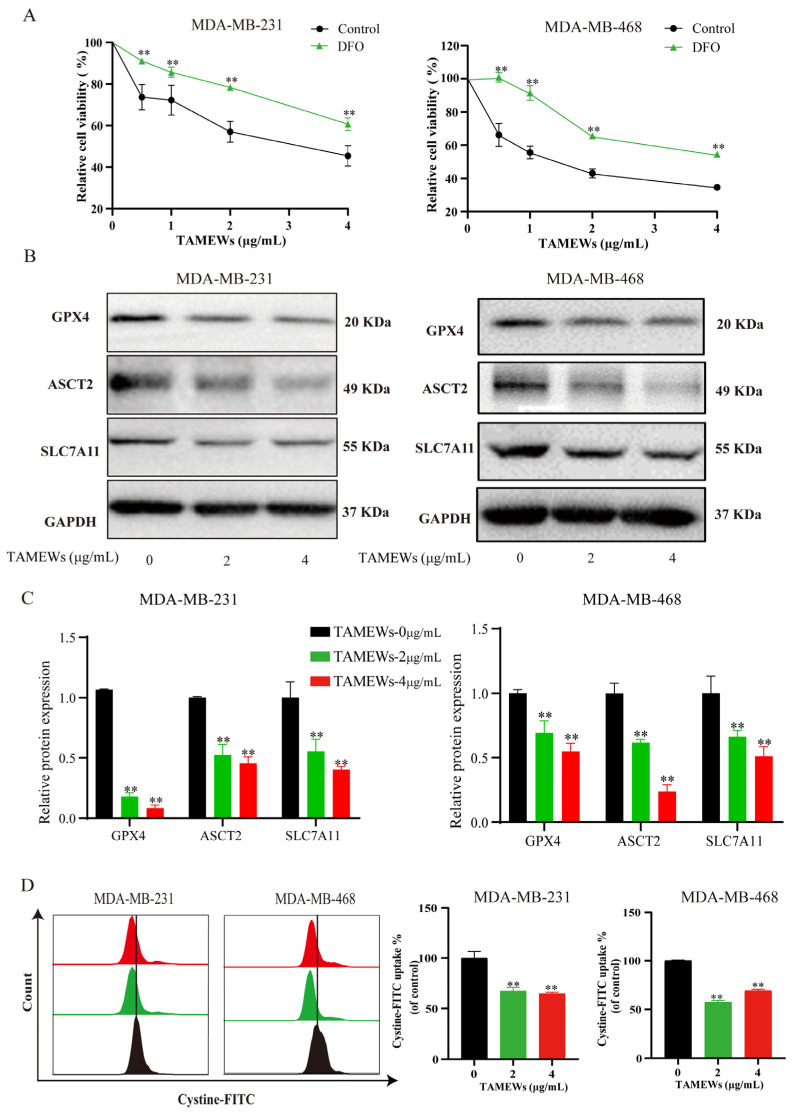
TAMEWs induce ferroptosis in TNBC cells by regulating amino acid metabolism. (**A**) Cells were treated with TAMEWs for 48 h after pre-treatment with the ferroptosis inhibitor DFO for 4 h. Relative cell viability was analyzed by the MTT assay. (**B**) Western blotting assay was performed to detect the expression of ASCT2, SLC7A11, and GPX4. (**C**) Statistical results of the relative protein expressions of ASCT2, SLC7A11, and GPX4. (**D**) Cystine uptake was detected by cystine-FITC probe and analyzed by flow cytometry. The data are expressed as mean ± SD (*n* = 3). ** *p* < 0.01 compared to the control groups.

The flow cytometry results showed that TAMEWs could induce the synthesis of lipid peroxides, and the accumulation of Fe^2+^ detected by the assay kit was higher than in the control group (Figure 6A–C). The levels of intracellular total GSH were decreased by TAMEW treatment (Figure 6D). All of the above suggest that TAMEWs could induce TNBC cell death by ferroptosis. Cellular lipid ROS were observed by fluorescence microscopy. The results showed that TAMEWs could enhance the fluorescence intensity of lipid ROS (Figure 6E). DFO was combined with TAMEWs to treat MDA-MB-231 and MDA-MB-468 cells. The inhibitory effect of TAMEWs on cell viability was reversed by DFO (Figure 7A). Western blotting results demonstrated that the expressions of the ferroptosis-related proteins SLC7A11, GPX4, and ASCT2 were decreased in TNBC cells after TAMEW treatment (Figure 7B,C). To evaluate the regulation by TAMEWs of the Xc^−^ system, the cystine-FITC uptake assay was performed by flow cytometry. The results revealed that TAMEWs could inhibit the uptake of cystine-FITC (Figure 7D). These collective findings confirmed that TAMEW-induced ferroptosis was regulated by amino acid metabolism.

### 2.5. TAMEWs Induce Ferroptosis via Regulation of p53 in the ASCT2-SLC7A11-GPX4 Axis

**Figure 8:**
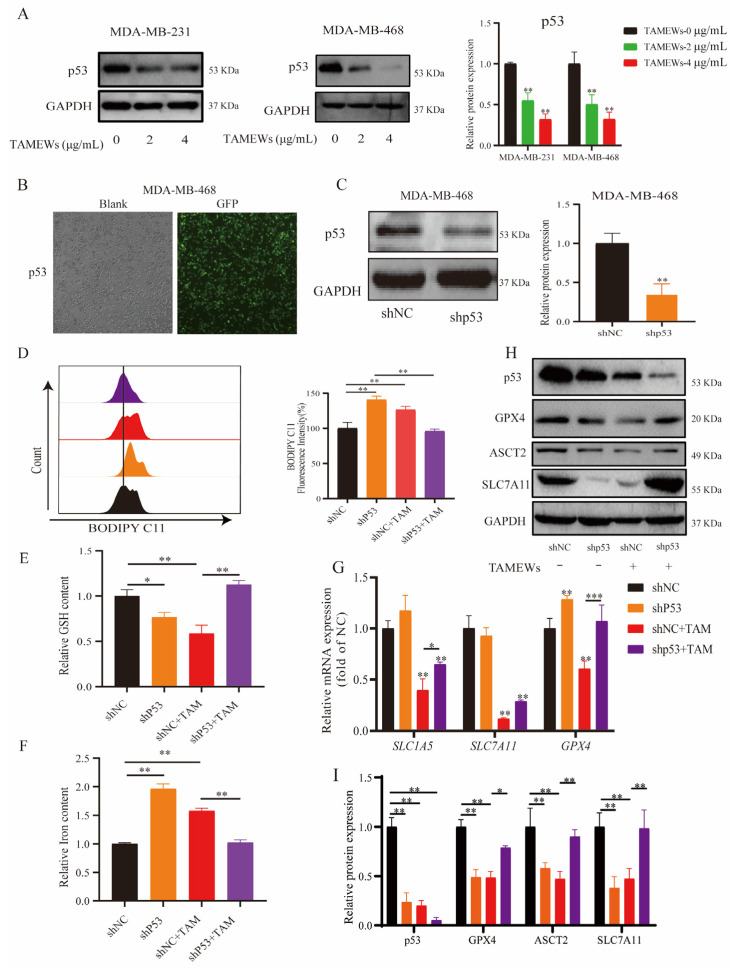
TAMEWs induce ferroptosis in TNBC cells via the p53-ASCT2-SLC7A11 axis. (**A**) Western blotting assay was performed to detect the expression of p53. (**B**,**C**) Lentivirus labeled with GFP was transfected and observed by a fluorescence microscope (200× magnification). (**D**) Lipid peroxidation was detected by the BODIPY-C11 (581/591) probe and analyzed by flow cytometry. (**E**) GSH level was detected by assay kit. (**F**) The total iron level was detected by assay kit. (**G**) The mRNA levels of *SLC1A5*, *GPX4*, and *SCL7A11* were measured by RT-PCR. (**H**) Western blotting assay was performed to detect the expression of p53, ASCT2, SLC7A11, and GPX4. (**I**) Statistical results of the relative protein expressions of p53, ASCT2, SLC7A11, and GPX4. The data are expressed as mean ± SD (*n* = 3). * *p* < 0.05, ** *p* < 0.01, *** *p* < 0.001 compared to the control groups.

Proteomics analysis revealed that the p53 signaling pathway changed significantly after TAMEWs’ intervention. Thus, we first verified the change of the p53 protein level with the intervention of TAMEWs, the results showed that TAMEWs could decrease the protein expression of p53 (Figure 8A). Secondly, to explore whether mutant p53 is involved in regulating the ASCT2-SLC7A11-GPX4 signaling axis with TAMEW intervention in TNBC, we knocked down the mutant p53 by lentivirus transfection in MDA-MB-468 (Figure 8B,C); the fluorescence of GFP and the Western blotting results showed that a stable transfected cell line was successfully established. Next, cells were treated with TAMEWs to detect intracellular lipid ROS, GSH, and iron levels. The results showed that after knocking down the mutant p53, the lipid ROS, GSH, and iron levels regulated by TAMEWs were significantly reversed (Figure 8D–F). RT-PCR results also showed that p53 knockdown could reverse the down-regulation of the expressions of *SLC1A5*, SLC7A11, and *GPX4* caused by TAMEW treatment (Figure 8G). The reduction in ASCT2, SLC7A11, and GPX4 expression induced by TAMEWs was rescued by p53 knockdown (Figure 8H,I).

### 2.6. TAMEWs Suppress Tumor Growth of TNBC In Vivo

**Figure 9:**
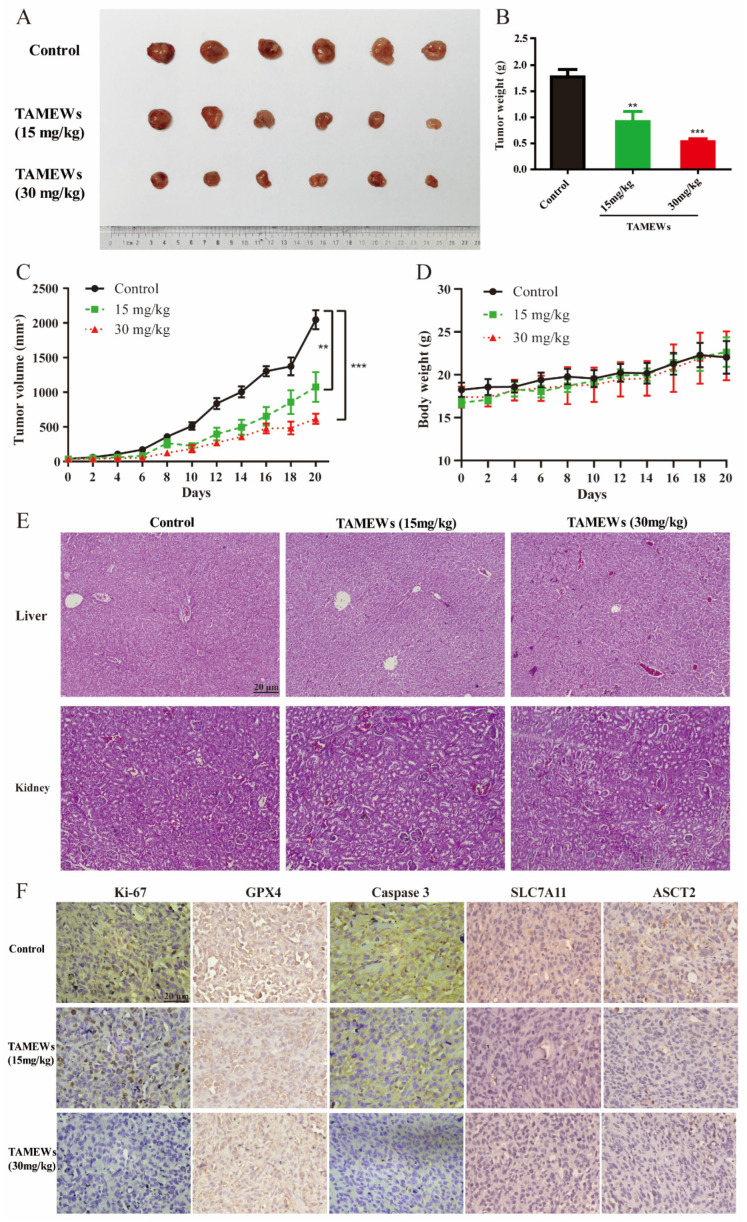
TAMEWs suppress tumor growth of TNBC in vivo. (**A**) Mice were sacrificed, and representative tumor images were then acquired. (**B**) The tumor mass was weighed. (**C**) Tumor size was recorded every other day by using a vernier caliper and calculated. (**D**) Average body weight was consecutively recorded. (**E**) Pathology of the liver and kidney of each group was observed by H&E staining. (**F**) Immunohistochemical analysis of Ki-67, GPX4, caspase 3, ASCT2, and SLC7A11. The data are expressed as mean ± SD (*n* = 6). ** *p* < 0.01, *** *p* < 0.001 compared to the control groups.

The xenograft model was established to further investigate the effects of TAMEWs in vivo. Tumor volume and weight in the TAMEW-treated groups were significantly decreased (Figure 9A–C). No significant difference in body weight was observed among the groups of mice (Figure 9D). HE staining showed that TAMEWs invoked no obvious injury to the kidney and liver (Figure 9E). Additionally, the immunohistochemical analysis revealed that TAMEWs decreased the expression of Ki-67, a sign of tumor proliferation (Figure 9F).

### 2.7. TAMEWs Inhibit TNBC In Vivo through Apoptosis and Ferroptosis

Finally, we detected the expression of caspase 3 in tumor tissues of nude mice. Caspase 3 expression in the TAMEW-treated groups was significantly lower than that in the control group, indicating that TAMEWs might induce TNBC cell death by inducing caspase 3-mediated apoptosis. Meanwhile, GPX4 expression was significantly decreased in the TAMEW-treated groups, suggesting that ferroptosis might also play an anti-TNBC role. SLC7A11 and ASCT2 were also decreased by TAMEW treatment in tumor tissue (Figure 9F), which indicated that TAMEWs could induce ferroptosis via ASCT2-SLC7A11-mediated amino acid metabolism. These collective findings demonstrated that TAMEWs could induce cell death by apoptosis and ferroptosis in vivo.

### 2.8. Discussion

Female BC has surpassed lung cancer as the leading cause of cancer. TNBC is the most serious subtype of BC, and current therapies are not effective. Therefore, it is necessary to develop drugs for the effective treatment of TNBC. Generally speaking, Solanaceae plants contain a large number of withanolides, a group of highly oxidized C28 ergosterol steroids, which usually have good anticancer activity [29]. As presented in our results, TAMEWs exhibited favorable anti-breast-cancer activity, especially against TNBC. Nowadays, RNA-seq and proteomics are becoming important tools to assist the anti-tumor effects of traditional Chinese medicine [30]. This study found that TAMEWs could induce TNBC cell death through apoptosis and ferroptosis by these two techniques.

Apoptosis, also known as type I cell death, represents a morphological pattern of programmed cell death. The main characteristic of apoptosis is the loss of MMP [31]. Many small-molecule drugs have been developed recently that can induce tumor cell apoptosis in cancers, including TNBC [32]. Mitochondria stimulation is a sign of early apoptosis [33]. Since the main pathological feature of mitochondrial dysfunction is changes in mito-ROS and MMP, this study evaluated whether TAMEWs could influence them in TNBC cells. In addition, our findings revealed that TAMEWs could increase the proportion of apoptotic cells (Figure 4A,B), and promote the cleavage of caspase 3 and PARP, but decrease the total protein expression of PARP (Figure 4C,D). The collective findings demonstrated that the TAMEWs induced caspase-dependent apoptosis through the mitochondria-mediated pathway in TNBC cells.

As reported, ROS accumulation might be the result of iron overloading [34]. Combined with the results of Section 2.2, we concluded that TAMEWs were likely to induce ferroptosis in TNBC cells. Ferroptosis is a regulated necrosis caused by a combination of iron toxicity, lipid peroxidation, and plasma membrane damage. Abnormal amino acid metabolism is also an essential event in ferroptosis [15]. TNBC cells depend on cystine for their survival and growth, likely due to the upregulated expression of cysteine transporters, such as SLC7A11 [35]. TNBC cells are sensitive to the inhibition of SLC7A11, which blocks cystine uptake, indicating the requirement of TNBC cells for cystine [35,36]. In addition, suppression of SLC7A11 activity in TNBC cells results in intracellular cysteine depletion, providing direct evidence that SLC7A11 may be highly responsive to extracellular amino acids [37]. In the present study, TAMEWs significantly induced lipid peroxidation, Fe^2+^ accumulation, and GSH scavenging (Figure 6A–D). Furthermore, TAMEWs suppressed the activity of system Xc^−^, indicated by the uptake of cystine-FITC (Figure 7D). Western blotting results showed that TAMEWs inhibit the expression of GXP4, SLC7A11, and ASCT2 (Figure 7B,C). Thus, TAMEWs may induce ferroptosis in TNBC cells through ASCT2-SLC7A11-mediated amino acid metabolism.

Mutations in p53 were found in 80% of TNBC cases. p53 gene mutation in TNBC is regarded as an important cause of chemotherapy resistance and poor prognosis [38]. Mutant p53 not only loses tumor-suppressive properties but also frequently gains tumor-promoting properties [39]. Current therapeutic strategies for targeting mutant p53 primarily involve reactivating the function of wild-type p53 and eliminating mutant p53 [40], and the present study focused on the elimination of mutant p53 in TNBC cells. The relationship between p53 and ferroptosis was reported in 2012, and further studies found that p53 played a key role in the regulation of the expression of ASCT2 and SLC7A11 [41,42]. Because there is little evidence that p53 is involved in the regulation of ASCT2-mediated glutamine metabolism [41], we explored the relationship between p53 and ASCT2, which has not been examined before. We demonstrated that TAMEWs down-regulated the expression of p53 (Figure 8A). In p53 knockdown TNBC cells, TAMEWs induced a reduction in GSH levels, and the elevation of total Fe^2+^ and lipid peroxidation were significantly reversed (Figure 8D–F), as well as inducing the protein down-regulation of ASCT2, SLC7A11, and GPX4 (Figure 8H,I).

Our data suggest the therapeutic potential of withanolides from *T. anomalum*. Ferroptosis mediated by amino acid metabolism may provide a new solution to treat TNBC. Though the present study has confirmed that TAMEWs could inhibit cystine uptake (Figure 7D), further study should focus on the cellular content of cystine and glutamine. Compared to the reported studies [41], this study first demonstrated that p53 knockdown could decrease the expression of ASCT2 and GPX4 in TNBC (Figure 8G–I). However, the specific mechanisms by which p53 rescues the TAMEW-induced down-regulation of GPX4 remain to be investigated. Furthermore, the role of amino acid metabolism in ferroptosis represents a crucial area that should be explored, since it may have an important role in tumor metabolic reprogramming.

## 3. Methods and Materials

### 3.1. Reagents

The 3-(4,5-Didmethylthiazol 3-(4,5-dimethylthiazol-2-yl)-2,5-diphenyltetrazolium bromide tetrazolium bromide (MTT) was purchased from Sigma-Aldrich (St. Louis, MO, USA). Cystine-fluorescein isothiocyanate (FITC) was purchased from Millipore (Birica, MA, USA). C11-BODIPY, deferoxamine (DFO), and carbobenzoxy-valyl-alanyl-aspartyl-[O-methyl]-fluoromethylketone (Z-VAD-FMK) were obtained from Selleck (Houston, TX, AABF-H, USA). Antibodies purchased from Cell Signaling Technology (Danfoss, MA, USA) included those against glyceraldehyde 3-phosphate dehydrogenase (GAPDH, #5174), β-actin (#4970), caspase 3 (#9662), cleaved caspase 3 (#9664), poly (ADP-ribose) polymerase (PARP, #9532), and ASCT2 (#8057s). Antibodies purchased from Abcam (Cambridge, UK) included those against GPX4 (ab125066), SLC7A11 (ab175186), and Ki-67 (ab16667). The ladder used for the western blotting (1610374) was purchased from Bio-Rad (Hercules, CA, USA).

### 3.2. Preparation of TAMEWs

The whole plant of *T. anomalum* (Franch. Et Sav.) Makino, collected as previously described [10], was concentrated to make it alcohol-free and then extracted with ethyl acetate. The ethyl acetate extracts were separated by silica gel column chromatography and octadecylsilyl (ODS) chromatography. Six withanolides, which made up the TAMEWs, were identified by ultra-high performance liquid chromatography-quadrupole time-of-flight mass spectrometry (UPLC-Q-TOF-MS/MS).

### 3.3. Cell Culture

All human TNBC and non-TNBC cell lines, MCF-10A, and HEK 293FT healthy mammary gland epithelial cell lines cells were kindly provided by the Shanghai Cell Bank of the Chinese Academy of Sciences (Shanghai, China). Cell lines were maintained in a DMEM/F12 medium containing 10% fetal bovine serum (FBS) and 1% penicillin/streptomycin. All reagents were purchased from Gibco (GrandIsland, NY, USA). HEK 293FT cells were cultured in a high-glucose Dulbecco’s modified Eagle’s medium (H-DMEM, Gibco, USA), supplemented with 10% FBS, 4 mM glutamine, 1% non-essential amino acids (Gibco, USA), and 1% sodium pyruvate (Gibco, GrandIsland, NY, USA). HEK 293FT cells were cultured in a high-glucose Dulbecco’s modified Eagle’s medium (H-DMEM, Gibco, GrandIsland, NY, USA), supplemented with 10% FBS, 4 mM glutamine, 1% non-essential amino acids, and 1% sodium pyruvate (all from Gibco) and 1% penicillin/streptomycin. Cells were cultured at 37 °C in a humidified 5% CO_2_ and 95% air atmosphere. Trypsin (0.25% *w*/*v*) with 0.2% (*w*/*v*) ethylene diamine tetra-acetate was used to dissociate and passage cells when cell confluence reached 80% to 90%.

### 3.4. Cell Viability Assay

The MTT assay was used to evaluate the cytotoxicity of TAMEWs on cell lines. Cells (3 × 10^3^ cells per well) were seeded in 96-well plates with complete medium and cultured overnight. The cells were then treated for 48 h with different concentrations of TAMEWs (0, 1.25, 2.5, 5, 10, 20, 50 μg/mL). To assess the influence of ferroptosis and apoptosis inhibitors (DFO and Z-VAD-FMK) on the inhibition of TNBC cells by TAMEWs, cells were seeded into plates as mentioned above, followed by the addition of DFO (100 μM), Z-VAD-FMK (10 μM), and the TAMEWs (0, 0.5, 1, 2, 4 μg/mL) for 48 h. After 48 h, 20 μL MTT (5 mg/mL) was added to each well, followed by incubation at 37 °C for 4 h. Dimethylsulfoxide was added to each well for 15 min, and the absorbance at 570 nm was measured by a multi-mode reader (Bio-Tek, Minneapolis, MN, USA).

### 3.5. RNA-Sequencing (RNA-Seq) and Proteomic Analysis

RNA-seq was performed by MultiSciences Biotech Co., Ltd. (Hangzhou, China). Proteomic analysis was completed by Dr. Maowei Ni, Zhejiang Province Cancer Hospital.

### 3.6. Mitochondrial Membrane Potential (MMP) Assay

Cells were seeded in a 6-well plate and cultured overnight. After a 12 h incubation with the TAMEWs (0, 2, 4 μg/mL), cells were incubated with 5,5′,6,6′-tetrachloro-1,1′,3,3′-tetraethylbenzimidazolyl-carbocyanine iodide (JC-1; Beyotime, Shanghai, China) working solution (10 μg/mL) for 30 min at 37 °C. When the MMP was high, JC-1 formed polymer aggregates (J-aggregates) within the mitochondrial matrix, resulting in red fluorescence. Conversely, JC-1 remained in a monomeric state at low MMP and emitted green fluorescence because it could not accumulate within the mitochondrial matrix. The JC-1 monomer and polymer had respective maximum excitation wavelengths of 514 and 585 nm, and maximum emission wavelengths of 529 and 590 nm. Fluorescence intensity was observed by fluorescence microscopy (Nikon, Tokyo, Japan).

### 3.7. Apoptosis Assessment

TNBC cells (5 × 10^4^ cells per well) were seeded in 6-well plates and incubated overnight. The different concentrations of TAMEWs (0, 2, 4 μg/mL) were added, and the cells were co-incubated for 24 h. The cells collected by centrifugation (1500 rpm, 5 min) were resuspended in a binding buffer. Annexin V-FITC and propidium iodide were added to each cell suspension and incubated in the dark for 15 min. Cell aggregates were removed by membrane filtration. The resulting suspension of individual cells was analyzed by flow cytometry using a CytoFLEX flow cytometer (Beckman, Brea, CA, USA), measuring Annexin V-FITC and PI fluorescence.

### 3.8. Detection of Mitochondrial ROS (Mito-ROS)

TNBC cells (5 × 10^4^ cells per well) were seeded in 6-well plates and incubated overnight. After a 24 h incubation with TAMEWs (0, 2, 4 μg/mL), the cells were collected and stained with a blank medium containing MitoSOX (5 μM) at 37 °C for 20 min. The mito-ROS level was evaluated by a CytoFLEX flow cytometer. At least 1 × 10^4^ events were recorded, and the mean values of fluorescence were quantified using FlowJo 10.6.2 software.

### 3.9. Lipid Peroxidation Detection

TNBC cells were collected after being treated with different concentrations of TAMEWs (0, 2, 4 μg/mL) for 24 h. C11-BODIPY (581/591) dye (Invitrogen, Carlsbad, CA, USA) was used to detect lipid peroxidation. Cells were harvested and washed three times using PBS. After incubating with C11-BODIPY (5 μM) at 37 °C for 20 min, the cells were collected by centrifugation (1500 rpm, 5 min, washed three times with PBS using the same centrifugation conditions, resuspended in a medium free of FBS and antibiotics, and analyzed using the CytoFLEX flow cytometer). At least 1 × 10^4^ events were recorded. Data were analyzed by FlowJo 10.6.2 software. The fluorescence intensity of C11-BODIPY (581/591) was observed by fluorescence microscopy (Nikon).

### 3.10. Intracellular Glutathione (GSH) Assay

TNBC cells were treated with different concentrations of TAMEWs (0, 2, 4 μg/mL) for 24 h and collected. The GSH/glutathione disulfide (GSSG) Assay Kit (Beyotime, Shanghai, China) was utilized following the manufacturer’s instructions. Total GSH and GSSG levels were assessed by scanning with a Cell Imaging multi-mode reader at a wavelength of 412 nm. The GSH concentration was determined by referencing an internal standard curve and then normalized to the total protein content measured from parallel plates.

### 3.11. Iron Assay

TNBC cells were collected after being treated with different concentrations of TAMEWs for 24 h. The relative content of iron was evaluated by an iron assay kit (APPLYGEN, Beijing, China) according to instructions. The absorption was detected at 550 nm. Data were normalized according to the protein content.

### 3.12. Cystine Uptake Assay

TNBC cells were collected after being treated with TAMEWs (0, 2, 4 μg/mL) for 24 h. The activity of system Xc^−^ was assessed using the cystine-FITC uptake assay. Briefly, the cells were exposed to the indicated drugs and then harvested by trypsinization. The cells were subsequently stained with a blank medium containing cystine-FITC (2 µM) at 37 °C for 30 min. The cells were washed with PBS and resuspended in 300 µL of fresh blank medium. Subsequently, the cells were analyzed using the CytoFLEX flow cytometer. At least 1 × 10^4^ events were recorded, and the mean values of fluorescence were quantified using FlowJo 10.6.2 software.

### 3.13. Plasmid and Cell Line Generation

Short hairpin RNA (shRNA) of the mutant-p53 gene was designed and synthesized by Genechem Co., Ltd. (Shanghai, China). The vector was hU6-MCS-CBh-gcGFP-IRES-puromycin. To generate the lentiviruses, third-generation viral packaging plasmids, and the lentiviral construct were transfected into HEK293FT cells using Lipofectamine. The cells were incubated with the viral supernatant and 0.6 µg/mL Polybrene (Millipore, Birica, MA, USA) before selection with 1 µg/mL puromycin (Invitrogen).

### 3.14. Quantitative Real-Time PCR Analysis

Cells were plated into a 6-well plate and then exposed to TAMEWs. Total RNA from TNBC cells was extracted by TRIzol reagent (Invitrogen). Equal amounts of RNA were reverse transcribed using a cDNA synthesis kit (Monad, Suzhou, China). Quantitative real-time PCR was performed using SYBR Green PCR Master Mix (Bio-Rad, USA) according to the manufacturer’s protocol. The primers used for qPCR were as follows: GPX4-F: 5′-AGAGATCAAAGAGTTCGCCGC-3′; GPX4-R: 5′-TCTTCATCCACTTCCACAGCG-3′; SLC1A5-F: 5′-TCATGTGGTACGCCCCTGT-3′; SLC1A5-R: 5′-GCGGGCAAAGAGTAAACCCA-3′; SLC7A11-F: 5′-TCTCCAAAGGAGGTTACCTGC-3′; SLC7A11-R: 5′-AGACTCCCCTCAGTAAAGTGAC-3′; GAPDH-F: 5′-GGGAGCCAAAAGGGTCATCA-3′; GAPDH-R: 5′-TCTTCATCCACTTCCACAGCG-3′. The relative target mRNA levels were determined using the 2^−(ΔΔCt)^ method and normalized against the GAPDH mRNA levels.

### 3.15. Western Blotting

The collected cells were lysed with RIPA buffer supplemented with phenylmethylsulfonyl fluoride. Equivalent amounts of protein were separated by SDS-PAGE and transferred to polyvinylidene difluoride membranes (Millipore, Birica, MA, USA). The membranes were blocked with 5% non-fat milk and incubated overnight at 4 °C with the specified primary antibodies, followed by incubation with the corresponding secondary antibodies. The expression of the target proteins was finally detected using the ECL detection system (Bio-Rad).

### 3.16. Tumor Xenograft

The animal studies were approved by the Institutional Animal Care and Use Committee of Zhejiang Chinese Medical University (No. 20210927-26) and were performed in accordance with the guidelines proposed by the Laboratory Animal Research Center of Zhejiang Chinese Medical University. Four-week-old athymic BALB/c female nude mice (nu/nu) were purchased from the Shanghai Experimental Animal Center (Shanghai, China). MDA-MB-231 cells (2.5 × 10^6^) were mixed with serum-free medium and injected into the bilateral flank of the nude mice. When the tumor volume reached 50 mm^3^, mice were treated either with vehicle or TAMEWs (15 and 30 mg/kg, respectively) every two days. Each group included six mice. Tumor size and body weight were monitored daily. Tumor volume was calculated as (length × width^2^)/2. After 14 days of treatment, mice were sacrificed and tumors, kidneys, and livers were obtained and analyzed by hematoxylin-eosin (H&E) staining and immunohistochemistry (IHC), as described next.

### 3.17. HE Staining and IHC

The obtained tissues were fixed with paraformaldehyde and embedded in paraffin, and sections 4 µm in thickness were cut. The sections were deparaffinized and dehydrated. Hydrogen peroxide (3%) in methanol followed by washes with PBS was used to block endogenous peroxidases. Normal bovine serum (5%) in PBS was used to prevent non-specific binding. HE staining was performed according to the manufacturer’s instructions to evaluate the liver and kidney toxicity of the TAMEWs. IHC for Ki-67, caspase 3, GPX4, ASCT2, and SLC7A11 was performed on tumor sections. Sections were placed on slides and put in a steamer. Hydrogen peroxide (3%) in methanol followed by washes with PBS was used to block endogenous peroxidases. Antigen repair was then performed with a sodium citrate buffer. The sections were incubated with a primary antibody against Ki-67 (1:200 dilution, Abcam), caspase 3 (1:200, Cell Signaling Technology, USA), GPX4 (1:100, Abcam), ASCT2 (1:400, CST), and SLC7A11 (1:200, Abcam) overnight at 4 °C, then incubated in the presence of horseradish peroxidase-conjugated secondary antibody, stained with 3,3n incubated, and counterstained with hematoxylin at room temperature. All slides were observed by bright-field microscopy at 40× magnification (ZEISS, Jena, Germany).

### 3.18. Statistical Analysis

All data were analyzed by GraphPad Prism 8.3. (GraphPad, San Diego, CA, USA). Data are expressed as mean ± standard deviation. A comparison of the two groups was performed using an unpaired Student’s *t*-test. One-way analysis of variance was used to determine the statistical difference when data were in a normal distribution. Dunnett’s test was performed for a variance that was not normally distributed. A *p*-value < 0.05 was considered statistically significant. All experiments were repeated at least three times.

## 4. Conclusions

TAMEWs from *T. anomalum* could be effective against TNBC. TAMEWs induced cell death through apoptosis via mitochondrial dysfunction and ferroptosis mediated by the p53-ASCT2-SLC7A11 axis. We hypothesize that *T. anomalum* has great potential for the treatment of TNBC.

## Data Availability

The datasets are available from the corresponding author upon reasonable request.

## Supplementary Materials

The following supporting information can be downloaded at: https://www.mdpi.com/article/10.3390/molecules29081838/s1, Figure S1: Structures and analysis of TAMEWs.
