# Supplementary material for: Novel Withanolides from Tubocapsicum anomalum Suppress Triple-Negative Breast Cancer by Triggering Apoptosis and p53-ASCT2-SLC7A11-Mediated Ferroptosis
Source: Molecules. 2024 Apr 18;29(8):1838. doi: 10.3390/molecules29081838 (PMC11052464; doi:10.3390/molecules29081838)
Supplement: Supplementary file 1 [file molecules-29-01838-s001.zip › molecules-2896624-supplementary.pdf]

Novel Withanolides from *Tubocapsicum anomalum* Suppress Triple-Negative Breast Cancer by Triggering Apoptosis and p53-ASCT2-SLC7A11-mediated ferroptosis

Lili Huang [1], Yingying Wei [1], Maowei Ni [2], Hongtao Hu [1], Luyi Xi [1], Chen Wang [1], Zhihui Zhu [1], Bo Yang [1,*] and Huajun Zhao [1,3,*]

[1] School of Pharmaceutical Sciences, Zhejiang Chinese Medical University, Gaoke Rd., Hangzhou 311402, China
[2] The Cancer Hospital of the University of Chinese Academy of Sciences (Zhejiang Cancer Hospital), Institute of Basic Medicine and Cancer (IBMC), Chinese Academy of Sciences, Hangzhou 310022, China
[3] Academy of Chinese Medical Sciences, Zhejiang Chinese Medical University, Binwen Rd., Hangzhou 310053, China
* Correspondence: ybtcm@zcmu.edu.cn (B.Y.); zhj@zcmu.edu.cn (H.Z.); Tel.: +86-0571-61768535 (B.Y.); +86-0571-61768535 (H.Z.)

Suppl. Figure S1

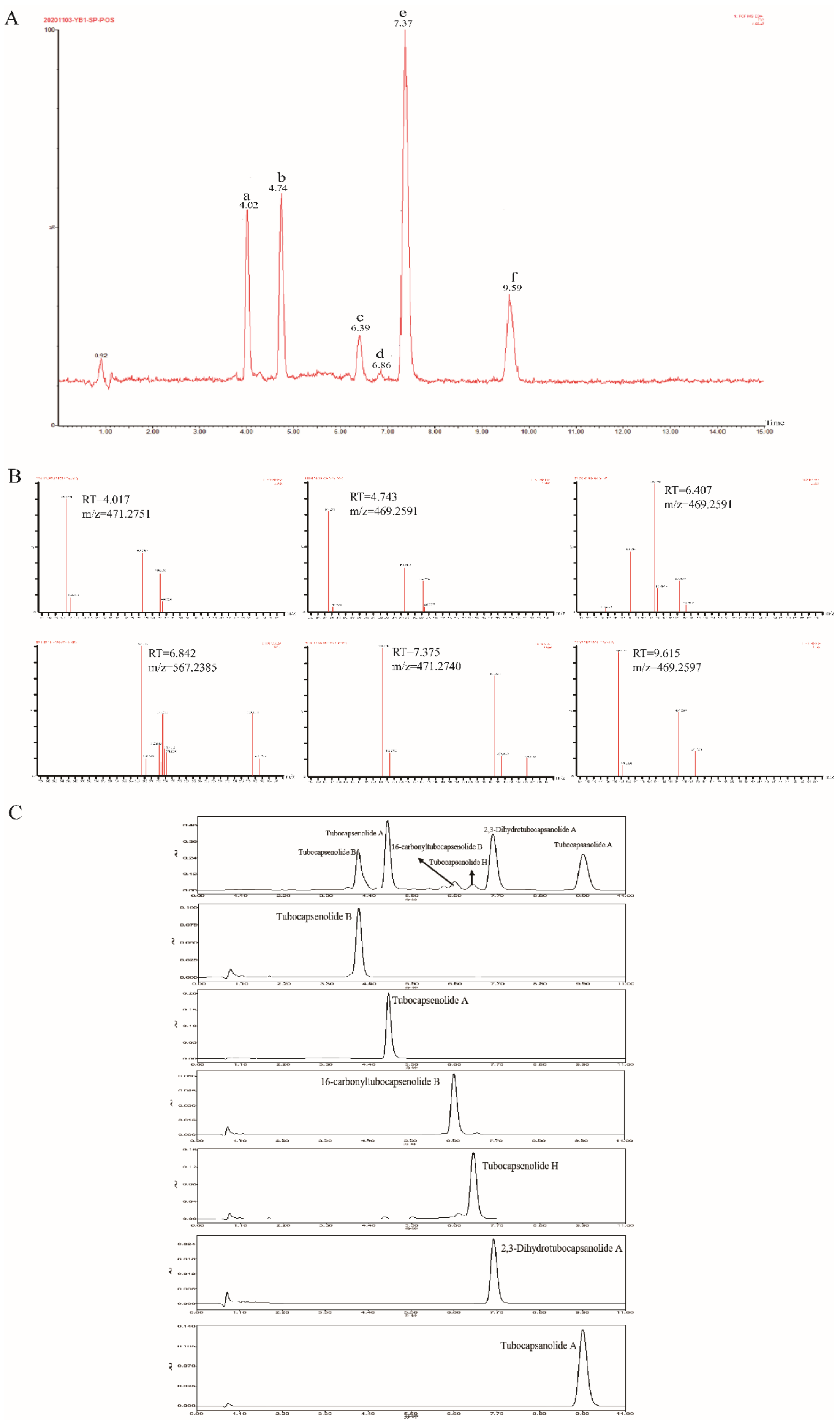
A
20201103-YB1-SP-POS
a 4.02
b 4.74
c 6.39
d 6.86
e 7.37
f 9.59
0.92
Time
B
RT=4.017 m/z=471.2751
RT=4.743 m/z=469.2591
RT=6.407 m/z=469.2591
RT=6.842 m/z=567.2385
RT=7.375 m/z=471.2740
RT=9.615 m/z=469.2597
C
Tubocapsenolide A
Tubocapsenolide B
16-carbonyltubocapsenolide B
Tubocapsenolide H
2,3-Dihydrotubocapsanolide A
Tubocapsanolide A

Figure S1. (related to figure 1) Structures and analysis of TAMEWs. (A) Total ions chromatogram of TAMEWs. IUPAC names are as follows: **a**: (2R,6S,7R,9R,14R,15S)-15-[(1S)-1-(4,5-dimethyl-6-oxo-3,6-dihydro-2H-pyran-2-yl)ethyl]-6,14-dihydroxy-2,15-dimethyl-8-oxapentacyclo[9.7.0.0^{2,7}.0^{7,9}.0^{12,16}]octadec-12(16)-en-3-one; **b**: (2R,6S,7R,9R,14R,15S)-15-[(1S)-1-(4,5-dimethyl-6-oxo-3,6-dihydro-2H-pyran-2-yl)ethyl]-6,14-dihydroxy-2,15-dimethyl-8-oxapentacyclo[9.7.0.0^{2,7}.0^{7,9}.0^{12,16}]octadeca-4,12(16)-dien-3-one ; **c**: (1S,2R,6S,7R,9R,11R,14R,15S)-15-[(1S)-1-[(2R)-4,5-dimethyl-6-oxo-3,6-dihydro-2H-pyran-2-yl]ethyl]-6,14-dihydroxy-2,15-dimethyl-8-oxapentacyclo[9.7.0.0^{2,7}.0^{7,9}.0^{12,16}]octadec-12(16)-en-3-one; **d**: (1S,2R,6S,7R,9R,11S,12S,14R,16S,17S)-16-[(1R)-1-[(2R)-4,5-dimethyl-6-oxo-3,6-dihydro-2H-pyran-2-yl]ethyl]-6-hydroxy-2,17-dimethyl-8,15-dioxahexacyclo[9.8.0.0^{2,7}.0^{7,9}.0^{12,17}.0^{14,16}]nonadecan-3-one; **e**: (1R,2S,6S,7R,10R,12S,17S,21R)-6-[(1S)-1-[(2R)-4,5-dimethyl-6-oxo-3,6-dihydro-2H-pyran-2-yl]ethyl]-7,21-dihydroxy-1,6-dimethyl-16-oxa-13-thiapentacyclo[10.8.1.0^{2,10}.0^{5,9}.0^{17,21}]henicosa-5(9),18-dien-20-one; **f**: (1S,2R,6S,7R,9R,11S,12S,14R,16S,17S)-16-[(1R)-1-[(2R)-4,5-dimethyl-6-oxo-3,6-dihydro-2H-pyran-2-yl]ethyl]-6-hydroxy-2,17-dimethyl-8,15-dioxahexacyclo[9.8.0.0^{2,7}.0^{7,9}.0^{12,17}.0^{14,16}]nonadec-4-en-3-one. (B) The secondary mass spectrometry fragment data of the six withanolides. (C) UPLC analysis of TAMEWs with standards.
